# Sagittal Alignment Correction in Single-Level Minimally Invasive Transforaminal Interbody Fusion with Unilateral vs. Bilateral Facetectomy

**DOI:** 10.3390/jcm14217595

**Published:** 2025-10-26

**Authors:** Sergej Telentschak, Eva Fruechtl, Moritz Perrech, Moritz Lenschow, Niklas von Spreckelsen, Dierk-Marko Czybulka, Roland Goldbrunner, Volker Neuschmelting

**Affiliations:** 1 Department of General Neurosurgery, Centre of Neurosurgery, Faculty of Medicine and University Hospital, University of Cologne, Kerpener Str. 62, 50937 Köln, Germany; eva.fruechtl96@t-online.de (E.F.); moritz.perrech@uk-koeln.de (M.P.); moritz.lenschow@uk-koeln.de (M.L.); nspreckelsen@wkk-hei.de (N.v.S.); dierk-marko.czybulka@uk-koeln.de (D.-M.C.); roland.goldbrunner@uk-koeln.de (R.G.); 2 Department of Neurosurgery, Westkuestenklinikum, Esmarchstraße 50, 25746 Heide, Germany

**Keywords:** transforaminal lumbar interbody fusion (TLIF), minimally invasive surgery (MIS), unilateral facetectomy (UF), bilateral facetectomy (BF), segmental lordosis

## Abstract

**Objective:** Bilateral facetectomy (BF) within minimally invasive transforaminal lumbar interbody fusion (MI-TLIF) remains debated regarding its advantages over unilateral facetectomy (UF) in restoring segmental lordosis, addressing spondylolisthesis and decompressing both neural foramina. The evidence is limited. We sought to determine the benefits of contralateral facetectomy on radiographic and clinical outcomes. **Methods:** We conducted a single-center retrospective analysis on patients with lumbar degenerative disease who underwent single-level percutaneous instrumentation and MI-TLIF with either UF or BF. Plain radiographs, CT and MRI were utilized for comparative radiographic analysis. Various intraoperative and clinical parameters were evaluated to assess surgical effort and clinical outcomes. **Results:** We included 81 UF and 23 BF cases; complete radiological data were available for 27 and 13 patients, respectively. Both techniques demonstrated a comparable increase in segmental lordosis (UF 2.1° ± 5.3° vs. BF 4.3° ± 5.4°, *p* > 0.1), which is below the study’s minimum detectable effect (MDE ≈ 5.1° at 80% power). Spondylolisthesis reduction was similar, with UF achieving a mean of 2.8 ± 2.2 mm and BF 2.4 ± 1.9 mm (*p* > 0.1). Mean posterior disc height did not differ significantly between groups (*p* > 0.1). The mean intraoperative blood loss was significantly higher with BF (803 ± 347 mL) compared to UF (437 ± 207 mL, *p* < 0.001). The mean duration of surgery was significantly longer for BF (240 ± 48 min) compared to UF (197 ± 37 min, *p* = 0.001). **Conclusions:** This study found no evidence of a large advantage of BF over UF in restoring segmental lordosis, spondylolisthesis and posterior disc height in monosegmental MI-TLIF surgery. Given the higher blood loss and longer operative time observed with BF, its use should be selective for specific indications.

## 1. Introduction

Transforaminal lumbar interbody fusion (TLIF) is a widespread standard procedure for treating degenerative segment disease, offering several advantages over alternative techniques in spinal fusion surgery [1,2,3,4,5]. TLIF has demonstrated favorable clinical outcomes, high fusion rates and robust mechanical stability, while providing an effective spinal canal and ipsilateral neural foramen decompression through unilateral facetectomy (UF) [3,6]. Bilateral facetectomy (BF) additionally facilitates a direct decompression of the contralateral neural foramen and its associated nerve root. However, there has been an ongoing debate regarding the superiority of BF over UF in restoring sagittal and coronal alignment, as well as the extent of additional surgical effort required for either technique: conventional open TLIF and minimally invasive TLIF (MI-TLIF) [7,8,9].

In theory, BF is believed to enable a larger interbody graft via a more extensive release, thereby improving indirect decompression of the contralateral neural foramen, and combined with a more comprehensive dorsal decompression, this results in an increased segmental lordosis angle, further enhanced by a dorsal segmental compression maneuver.

Previous finite element studies and radiographic investigations indicate that posterior bone resection combined with bilateral facet removal can effectively restore segmental and lumbar lordosis, thereby correcting sagittal imbalance and potentially enhancing clinical outcomes while mitigating further degeneration [10,11]. Some authors consider bilateral facetectomy a crucial element for enhancing lordosis during TLIF, akin to a posterior column osteotomy. This approach mitigates the rigidity imposed by the facet joints and enhances visualization of anterior structures for any required release [12,13,14]. Other researchers have reported significantly improved clinical outcomes following the removal of both facet joints during conventional open TLIF, suggesting that they may be a source of pain and should be removed in eligible patients [15,16].

Clinical studies providing a direct comprehensive comparison of BF with UF are scarce and report controversial results. Furthermore, a biomechanical cadaveric comparison of both techniques in the MI-TLIF procedure revealed divergent radiological outcomes.

The current literature provides a comprehensive background on TLIF, but direct evidence contrasting unilateral versus bilateral facetectomy (UF vs. BF) in MIS-TLIF remains limited and partly conflicting, particularly regarding segmental lordosis restoration, spondylolisthesis reduction and the need for bilateral foraminal decompression, as well as the overall benefit–risk profile [3,6,14]. Therefore, we aimed to compare radiographic and clinical outcomes after single-level MIS-TLIF using BF versus UF. Our primary objective was to assess segmental lordosis; secondary objectives included spondylolisthesis correction, bilateral foraminal decompression, perioperative burden (operative time and blood loss) and complications, to delineate the clinical and radiological benefit–risk balance of BF relative to UF.

## 2. Materials and Methods

### 2.1. Patient Selection and Clinical Analysis

We conducted a retrospective analysis of our patients with lumbar segmental degenerative disease who underwent single-level MI-TLIF surgery and dorsal percutaneous navigated instrumentation, screening 171 patients with any TLIF surgery between 2017 and 2021 at our spine center, and reviewed their perioperative clinical records, excluding 67 patients with multilevel, adjacent-level, traumatic, spondylodiscitis and open TLIF cases.

Patient demographic and clinical information including age, gender, body mass index (BMI), general health status according to ASA grade and general medical conditions were recorded (Table 1). All patients were followed up at least 6 weeks postoperatively, in order to detect early postoperative complications.

The surgical indications were lower back pain and leg pain associated with a single-level lumbar or lumbosacral degenerative or isthmic spondylolisthesis of low grade (grade I/II) with segmental translational instability of ≥4 mm or angular motion of ≥10° on preoperative flexion and extension radiographs. Further indications were recurrent disc herniations, symptomatic foraminal stenosis or erosive intervertebral osteochondrosis (Table 2).

The bilateral approach was chosen in cases with severe loss of lordosis, bilateral symptomatic spondylotic neuroforaminal stenosis or locked facets. The side of facetectomy was decided according to preoperative clinical symptoms or radiological examination.

Surgical effort and burden were assessed by intraoperative blood loss, surgery duration, intraoperative adverse events, postoperative complications, pain levels according to the numeric rating scale (NRS), the WHO pain ladder and hospital duration (Table 2).

Because this was a retrospective chart review, some baseline variables (e.g., BMI and ASA class) were not consistently recorded in the index admission or were documented in external preoperative reports not accessible in our archive. Some postoperative variables (e.g., pain levels according to the NRS) were incompletely documented in our hospital’s electronic medical system. We report the effective sample sizes for each table entry. For inferential analyses, we used available-case data; adjusted models were fitted on complete cases with the analysis-specific N reported. As sensitivity analyses, we included a missing-indicator term for any baseline covariate and performed best-/worst-case bounds for the binary outcomes. These checks yielded the same qualitative conclusions.

### 2.2. Surgical Technique

**Figure 1:**
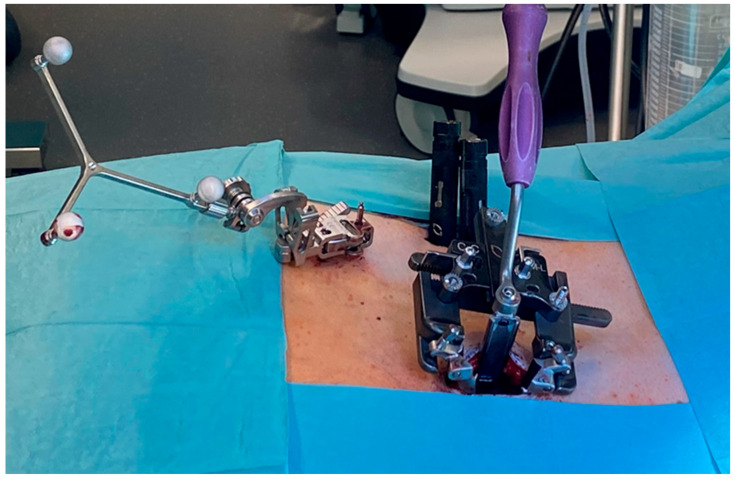
Intraoperative photograph of the navigated dorsolateral percutaneous transpedicular instrumentation in L4/5 with an MIS-TLIF over the right side facetectomy showing a navigation reference array firmly attached to the patient’s iliac crest (left on the picture), percutaneous pedicle screw head extenders (on the right in the background of the picture) and an MIS retractor with two valves attached to the right side pedicle screws of L4/5 (in the foreground of the picture) enabling a distraction of the segment; the third retractor valve enables a sufficient medial muscle mass retraction, facilitating a wide decompression of the spinal canal and undercutting of the opposite side.

First, percutaneous pedicle screw instrumentation was performed with the assistance of spinal neuronavigation. On the MI-TLIF side, the spine was approached through a 3–4 cm long incision in the area about 3–4 cm from the midline of the back along the retractor valves attached to the screw heads (Figure 1). The multifidus and longissimus dorsi muscles were pulled off to reach the facet joint and the lamina of the vertebral arch. The inferior and superior articular facets were removed by a chisel. Then, under microscopic visualization, hemilaminotomy and excision of the ligamentum flavum were performed using a high-speed drill and punches exposing the spinal canal with the dura mater and traversing nerve root as well as the neural foramen with the exiting nerve root. A contralateral undercutting with a decompression of contralateral recess was performed if necessary. Subsequently, the discectomy was performed, and the interbody spaces were carefully extended using a shaver, then curettage of the endplate finalized the preparation of the superior and inferior lumbar endplates for fusion. The disc space and PEEK-Cage were filled with collected bone fragments, then the banana-shaped and bone-filled PEEK-Cage was inserted into the disc space. All cages were lordotic with an angle of 8°. Following reduction and compression, the inserted rods were locked with the setscrews on both sides.

In the bilateral facetectomy group, the spine was bilaterally approached through two abovementioned 3–4 cm long incisions in the area about 3–4 cm from the midline of the back, and at least one facetectomy with a decompression of the exiting nerve root was performed on the contralateral side in the same way, as described above. Subsequently, rods were inserted and locked with the setscrews after reduction and compression on both sides.

### 2.3. Radiological Analysis

**Figure 2:**
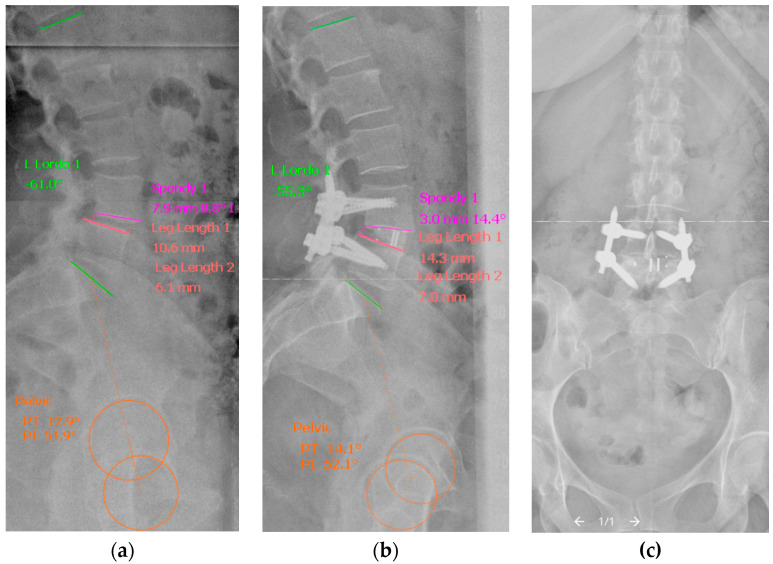
Representative plain radiographs illustrating the measurement of the segmental, regional and pelvic sagittal radiographic parameters by Surgimap^®^ (Version 2.3.2.1) on preoperative (**a**) and postoperative (**b**) standing lateral plain radiographs of the whole spine after MIS-TLIF and percutaneous pedicle screw fixation: segmental disc angle in degrees and spondylolisthesis in mm are measured by the “Spondy” tool; anterior and posterior disc height by the “LLength” tool; lumbar lordosis in degrees by the “LLordo” tool; and pelvic incidence, pelvic tilt and sacral slope in degrees by the “Pelvic” tool. A corresponding postoperative anterior–posterior projection of the lumbar spine (**c**).

Numerous radiographic parameters including segmental (segmental disc angle in degrees, spondylolisthesis, anterior and posterior disc height in mm) [8,17,18,19], regional spinal (lumbar lordosis in degrees) and pelvic sagittal parameters (pelvic incidence, pelvic tilt and sacral slope in degrees) [20] as well as global sagittal balance (sagittal vertical axis [21,22] in mm and odontoid–hip axis [23] in degrees) were measured on pre- and postoperative standing plain radiographs of the whole spine for comparing analysis by Surgimap^®^ (Version 2.3.2.1, Nemaris Inc., New York City, NY, USA) (Table 3, Figure 2). Preoperative CT and MRI of the lumbar spine were analyzed for radiological assessment of the Baastrup phenomenon and facet join degeneration according to a modified classification of Weishaupt et al. [17,24]. Indirect decompression of the contralateral neural foramen by the cage was estimated by means of posterior disc height (PDH) as a part of segmental parameters [8,18]. All measurements and gradings were performed as referenced. An independent reviewer, who was blinded to the applied surgical technique and the operating surgeons involved in the study, performed measurements of all radiographic parameters.

### 2.4. Statistical Analysis

Descriptive statistics are expressed as mean ± standard deviation, median (range), median (95% confidence interval) or counts with percentages, as appropriate. Categorical variables were compared by chi-square and Fisher’s exact tests, when appropriate. Continuous variables were tested for normal distribution using the Kolmogorov–Smirnov test, the Shapiro–Wilk test and visual inspection of Q-Q plots. Due to non-normal distribution, all continuous variables were compared using a Mann–Whitney U test for independent samples and a Wilcoxon test for dependent samples. Multinomial logistic regression was conducted for analysis of potential factors impacting on segmental lordosis, and homoscedasticity was assessed using White’s test and residual plots. Given the retrospective, fixed sample size for the primary radiological endpoint (segmental lordosis angle gain: n = 27 for EF and n = 13 for BF), we conducted a post hoc minimum detectable effect (MDE) analysis for two independent samples (two-sided α = 0.05, target power 80%). Numerical results are reported in the Results section. All calculations were performed using SPSS software (Version 30, IBM SPSS Statistics for Windows, Armonk, NY, USA). A *p*-value < 0.05 was considered statistically significant.

## 3. Results

We retrospectively identified 104 patients with suitable clinical records who suffered from symptomatic lumbar segmental degenerative disease and underwent single-level dorsal percutaneous navigated instrumentation and MI-TLIF surgery with either a UF (n = 81) or a BF (n = 23), including 13 BF patients and 27 UF patients who received suitable pre- and postoperative standing radiographs of the whole spine or at least the lumbar spine. Cross-sectional imaging (CT and MRI) was available for all 104 patients, but it generally cannot be used for perioperative comparison of sagittal alignment parameters due to well-known differences between supine and standing positions.

There was no significant difference found between the two groups in either patient demographics (patient age and gender) or health status measured by BMI, ASA grade and different general medical conditions (*p* > 0.1) (Table 1).

Regarding surgery-related characteristics, there was no significant difference found between the two groups in hospital duration, surgical wound pain levels, the need for pain killers at discharge or intraoperative and early postoperative adverse events (all *p* > 0.05). The distribution of the cage height (all cages with obliquity of 8°), cage position, operated segments, Bastrup phenomenon and degree of facet degeneration was not significantly different across both cohorts (UF vs. BF, all *p* > 0.05, see Table 2). The level of missing data was low to moderate across surgery-related, demographic and health-status-related variables (details in Table 1 and Table 2 footnotes). Patients with missing baseline data did not differ materially compared with those with complete data.

**Figure 3:**
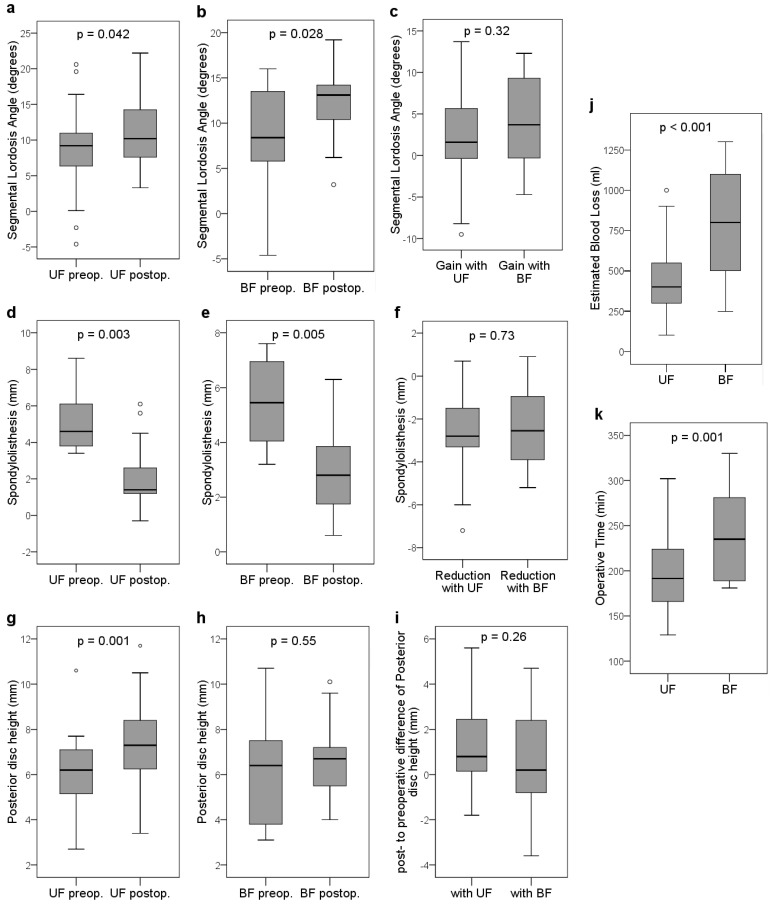
Dynamics of segmental radiographic and clinical parameters prior vs. after monosegmental MI-TLIF surgery with unilateral facetectomy (UF) vs. bilateral facetectomy (BF): gain in segmental lordosis (**a**–**c**) and spondylolisthesis reduction (**d**–**f**); comparison of the pre- and postoperative posterior disc height (PDH) with UF (**g**) an BF (**h**) as well as comparison of their mean differences (**i**); intraoperative blood loss (**j**) and surgery duration (**k**); ○ = outliers.

Both techniques showed a significant postoperative gain in segmental lordosis by a mean of 2.1° ± 5.3° (*p* = 0.042) in the UF and 4.3° ± 5.4° (*p* = 0.028) in the BF group (Table 3, Figure 3a,b). The significant lordosis correction was also supported by a significant gain in anterior disc height (ADH) by a mean of 2.8 mm (both *p* < 0.05), accompanied by less gain in posterior disc height (PDH) in both groups. The difference in magnitudes of segmental lordosis angle gain between both groups was not significant (*p* = 0.32) (Table 3, Figure 3c), but the post hoc assessment indicated an MDE of Cohen’s d = 0.97 for this primary radiological endpoint. Using the pooled SD (≈5.30°), this corresponds to an absolute difference of approximately 5.14°. For a non-parametric interpretation, this maps to a Mann–Whitney AUC ≈ 0.754 and Cliff’s δ ≈ 0.507, indicating adequate power to detect large but not small-to-moderate differences (two-sided α = 0.05; n = 27 vs. 13).

The spondylolisthesis was found to be significantly reduced by a mean of 2.8 ± 2.2 mm in the UF group (*p* = 0.003) as well as in the BF group by a mean of 2.4 ± 1.9 mm (*p* = 0.005) (Table 3, Figure 3d,e). There was no statistical difference in the reduction in spondylolisthesis between the two groups (*p* = 0.73) (Table 3, Figure 3f).

Posterior disc height (PHD), as a surrogate of indirect decompression of the contralateral neural foramen by the cage, showed a significant mean increase of 1.3 mm ± 1.9 mm after a TLIF with UF (*p* = 0.001) (Table 3, Figure 3g–i). In comparison, the BF cohort showed a minor non-significant mean PDH increase of 0.4 mm ± 2.6 mm (*p* = 0.55). Gain in PHD as well as AHD between UF and BF cohorts did not differ (both *p* > 0.1).

The mean intraoperative blood loss was significantly increased in the BF patients (803 ± 347 mL) compared to UF (437 ± 207 mL, *p* < 0.001) (Table 2, Figure 3j). The mean surgery duration was significantly longer with BF (240 ± 48 min) than with UF (197 ± 37 min, *p* = 0.001) (Table 2, Figure 3k).

In a multinomial logistic regression analysis, the Baastrup phenomenon, the grade of facet degeneration (grade 0 + 1 + 2 vs. grade 3 + 4), the bilateral facet resection, the cage position (ventral vs. middle) and the cage height (all 8° oblique) were not significantly associated with the gain in lordosis if comparing loss of lordosis (<0°) vs. low gain in lordosis (0–2°) and vs. distinct gain in lordosis (>2°) after surgery (all *p* > 0.05, see Table 4). Because heteroskedasticity was detected (White’s test, *p* < 0.05), these results should be interpreted with caution.

Regarding all intraoperative adverse events and early postoperative complications in total, there was no difference found between both groups in the chi-square test (all *p* > 0.1; for detail, see Table 5).

## 4. Discussion

With the exception of one metric, all evaluated segmental sagittal parameters improved significantly after surgery in both the UF and the BF cohort. The only exception was posterior disc height (PDH), which showed a small, non-significant increase after BF. Given that the MDE analysis for the primary radiological endpoint (segmental lordosis angle gain) corresponds to a large effect (Cohen’s d ≈ 0.97; ≈5.14°); analyses of secondary endpoints were considered exploratory and may be underpowered. For both primary and secondary outcomes, non-significant findings should be interpreted with caution, as smaller—yet potentially clinically meaningful—differences may not have been detectable. Regardless of this limitation, our radiographic results are consistent with previous retrospective studies—Min et al. [7] for MIS-TLIF and Tye et al. [16] for conventional open TLIF—in single-level degenerative disease, demonstrating no significant advantage of BF over UF for segmental lordosis, postoperative disc height, or spondylolisthesis reduction. By contrast, the biomechanical cadaver study by Snyder et al. [14] reported a radiographic advantage of BF; however, its ex vivo design likely fails to capture key intraoperative and in vivo factors, limiting the applicability of those findings to clinical practice.

The factors like Baastrup phenomenon, grade of facet degeneration, bilateral facet resection, cage position and cage height are discussed to impact the gain in the segmental lordosis. However, our multivariable model did not demonstrate such associations—likely limited by a heteroskedastic variance structure, small sample size and risk of overfitting, as reflected by wide confidence intervals. Accordingly, non-significant results should not be taken as evidence of no association; estimates are imprecise and require confirmation in larger, adequately powered cohorts.

Theoretically, resection of additional posterior elements should yield greater segmental lordosis. However, neither the cited MIS-TLIF [7] nor the cited open-TLIF study [16] demonstrated a superior lordosis gain with bilateral compared with unilateral facetectomy, nor did our data. Thus, we suppose that in standard TLIF procedures, bilateral facetectomy may not provide sufficient posterior column release to achieve a significant radiological increase in lordosis over unilateral facetectomy. Substantially greater correction likely requires more extensive posterior column osteotomies (e.g., Ponte), which are not the intent of standard TLIF and are even less feasible with MIS-TLIF.

The literature and expert opinions are divided regarding the contralateral foramen. Several authors report inadequate indirect contralateral foraminal decompression [7,25,26], while others maintain that unilateral TLIF (UF) can provide successful indirect decompression through interbody distraction/height restoration [8,27]. In our data, PDH—used as a surrogate for contralateral foraminal patency—showed a significant mean increase from pre- to postoperative (Figure 3g–i), consistent with effective indirect decompression at the group level. Nonetheless, individual PDH changes varied substantially, indicating that unilateral TLIF cannot ensure sufficient contralateral decompression in every case. We therefore consider direct decompression with BF a more reliable strategy for MIS-TLIF when bilateral symptomatic foraminal stenosis is present. This is in line with Hunt et al. [25], who reported a postoperative decrease in contralateral foraminal height, accompanied by reduced PDH, in 2.5% of unilateral open TLIF cases. As they suggest, increased segmental lordosis may precipitate decompensation of pre-existing, asymptomatic contralateral stenosis, warranting a larger interbody graft and consideration of direct contralateral decompression.

With respect to intraoperative burden, both estimated blood loss and operative time were significantly higher in our BF group, consistent with the additional, time-consuming contralateral exposure; this aligns with the findings of Min et al. [7] for MIS-TLIF. By contrast, in conventional open single-level TLIF, Tye et al. [16] reported no significant differences in blood loss or operative time between BF and UF. Kasis et al. [28] even observed shorter operative times with BF in an open approach despite wider exposure, hypothesizing that direct bilateral access enables rapid, complete disc space preparation. These discrepancies may reflect the low incremental effort in larger open exposures, where both facet joints are already accessible. Accordingly, intraoperative burden—particularly blood loss and operative time—should be considered when selecting the TLIF approach (MIS-TLIF with UF vs. MIS-TLIF with BF vs. open TLIF). For MIS-TLIF, we recommend BF for cases with symptomatic or at least radiographic evidence of relevant contralateral foraminal stenosis.

For completeness, measurements of global and regional spinal and pelvic sagittal alignment did not reveal relevant changes beyond the segmental parameters, consistent with a monosegmental correction by MIS-TLIF. The observed significant postoperative decrease in lumber lordosis in both cohorts was attributed to a temporary pain-related posture in the early postoperative phase.

Regarding intraoperative adverse events, ipsilateral dural tears were more frequent in the UF group (21%; 17/81) than in the BF group (8.7%; 2/23). Notably, no dural tears occurred on the side of the contralateral facetectomy in the BF cohort. This suggests that a two-sided approach to the canal may facilitate safer decompression.

Moreover, overall adverse events and complications did not increase in the BF cohort, even though mean intraoperative blood loss and operative time were significantly higher. This aligns with prior findings [7]. Because of the relatively small sample size, a meaningful stratification of events was not possible.

Limitations: The retrospective study design, small and unequal patient cohorts with different surgical indications and potential bias in intraoperative management between different surgeons cannot be excluded from the dataset. Given the small sample size of the radiological subgroup and an MDE consistent with detection of large effects only, statistical power was limited. Non-significant results should not be interpreted as equivalence; smaller effects may have escaped detection. Heterogeneously favored pre- and postoperative imaging modalities of different surgeons led to further limitations of radiologic comparability. The estimation of indirect decompression of the contralateral neural foramen was virtually limited by a surrogate parameter PDH. Due to a relatively short follow-up of 6–8 weeks after surgery, our emphasis in interpreting this study lies in discussing potential benefits and drawbacks of a specific technique in light of perioperative and early postoperative segmental parameters; long-term clinical and radiographic durability cannot be inferred from our data.

## 5. Conclusions

Our single-level MIS-TLIF data show no superiority of BF over UF for large effects on segmental sagittal alignment. Practically, BF should be used selectively—e.g., in cases with symptomatic bilateral high-grade foraminal stenosis and a clear need for direct contralateral decompression.

## Figures and Tables

**Table 1:** Patient demographics and health status.

Parameters	UF	BF	*p*	Available n for UF/BF *
Mean patient age (yrs)	64 ± 11	62.4 ± 10.6	0.46	81/23
Gender (f:m, %)	60:40	52:48	0.48	81/23
Mean BMI	29.6 ± 7	28.9 ± 5.7	0.56	80/23
Distribution of ASA grades (1:2:3, %)	6:67:27	17:52:31	0.22	78/23
General medical conditions, % (n):			>0.1	81/23
Diabetes mellitus	14.8 (12)	8.7 (2)		
Obesity	48.1 (39)	34.8 (8)		
Atherosclerosis	13.6 (11)	13 (3)		
Prior thrombosis *w*/*o* PE	7.4 (6)	4.3 (1)		
Smoking history	29.6 (24)	34.8 (8)		
Anticoagulant drugs	2.5 (2)	4.3. (1)		
COPD	12.3 (10)	8.7 (2)		

* The last column shows, for each variable, the number of patients with available data. The total enrolled sample sizes were 81 for BF and 23 for UF.

**Table 2:** Clinical and surgery-related characteristics. * Significant value *p* < 0.05.

Parameters	UF	BF	*p*	Available n for UF/BF *
Previous spinal surgery at treated or adjacent level, % (n)	38.3 (31)	21.7 (5)	0.21	81/23
Diagnosis related to surgery, % (n):			0.005 *	81/23
Degenerative spondylolisthesis	74.1 (60)	65.2 (15)		
Recurrent disc herniation	7.4 (6)	-		
Isthmic spondylolisthesis	4.9 (4)	30.4 (7)		
Symptomatic foraminal stenosis	7.4 (6)	4.4 (1)		
Osteochondrosis	6.2 (5)	-		
Bastrup phenomenon, % (n)	57 (45)	39.1 (9)	0.13	79/23
Grade of facet joint degeneration [17,18], % (n)			0.09	79/23
Grade 2	20.3 (16)	34.8 (8)		
Grade 3	50.6 (40)	56.5 (13)		
Grade 4 (locked facets)	29.1 (23)	8.7 (2)		
Need for pain killers at admission (WHO grade), median (range)	2 (1–3)	1 (1–3)	0.29	41/16
Median postop. wound pain levels (NRS)	2 (0–8)	2.5 (0–4)	0.66	63/14
Postop. pain at motion and ambulation, median (range)	2 (0–10)	3.5 (0–7)	0.24	63/14
Need for pain killers at discharge (WHO grade), median (range)	3 (1–3)	3 (1–3)	1	81/23
Hospital duration (d)	6.1 ± 2.6	6.0 ± 3.1	0.16	81/23
Operated segments, % (n)			>0.1	81/23
L 1/2	0	0		
L 2/3	1.2 (1)	4.3 (1)		
L 3/4	8.7 (7)	0		
L 4/5	75.3 (61)	74 (17)		
L 5/S1	14.8 (12)	21.7 (5)		
Median cage height (all lordotic 8°), mm	11 (9–14)	11 (9–12)	0.73	81/23
Cage position, % (n): Ventral	59.5 (47)	56.5 (13)	0.79	79/23
Middle	40.5 (32)	43.5 (10)		
Medial intraoperative blood loss (mL)	437 ± 207	803 ± 347	<0.001 *	81/23
Medial operative time (min)	197 ± 37	240 ± 48	0.001 *	

* The last column shows, for each variable, the number of patients with available data. The total enrolled sample sizes were 81 for BF and 23 for UF.

**Table 3:** Radiographic characteristics. Δ = difference between post- and preoperative values. * Significant value *p* < 0.05.

Parameters	UF (n = 27)			BF (n = 13)			*p*
	Preop.	Postop.	*p*	Preop.	Postop.	*p*	
Segmental Spinal Sagittal Parameters							
Segmental lordosis angle (SLA), °	8.9 ± 5.6	11 ± 4.8	0.042 *	7.98 ± 6.6	12.3 ± 4.6	0.028 *	
SLA gain (Δ post- to preop.), °	2.1 ± 5.3			4.3 ± 5.4			0.32
Spondylolisthesis (SL), mm	5.1 ± 1.6	2.3 ± 1.9	0.003 *	5.4 ± 1.6	2.98 ± 1.8	0.005 *	
SL reduction (Δ post- to preop.), mm	−2.8 ± 2.2			−2.4 ± 1.9			0.73
Posterior disc height (PDH), mm	5.96 ± 1.8	7.3 ± 1.97	0.001 *	6.2 ± 2.3	6.6 ± 1.9	0.55	
Δ PDH, mm	1.4 ± 1.9			0.4 ± 2.6			0.26
Anterior disc height (ADH), mm	11.3 ± 3.1	14.1 ± 2.8	0.000 *	10.4 ± 4.3	13.2 ± 2.4	0.033 *	
Δ ADH, mm	2.8 ± 2.8			2.8 ± 4			0.65
Regional Spinal Sagittal Parameters							
Lumbar lordosis angle (LLA), °	51.6 ± 13.9	48.8 ± 12.3	0.049 *	53.8 ± 17.3	48.2 ± 24.5	0.039 *	
Δ LLA, °	−2.8 ± 8.9			−5.5 ± 9.4			0.42
Pelvic Sagittal Parameters							
Pelvic incidence (PI), °	55.8 ± 9.2	55.6 ± 9.2	0.38	57.8 ± 9.5	58.2 ± 9.6	0.39	
Δ PI, °	−0.2 ± 1.1			0.4 ± 2.5			0.29
Sacral slope (SS), °	37.2 ± 7.1	35.9 ± 5.98	0.14	39.4 ± 7.9	38.7 ± 7.4	0.28	
Δ SS, °	−1.3 ± 4.5			−0.7 ± 3.8			0.69
Pelvic tilt (PT), °	18.7 ± 8.3	19.7 ± 6.9	0.13	18.5 ± 6.7	19.5 ± 5.7	0.38	
Δ PT, °	1.1 ± 4.7			0.96 ± 4.5			0.9
Global Spinal Sagittal Parameters							
Sagittal vertical axis (SVA), mm	41.3 ± 42.9	49.1 ± 34.4	0.07	34.1 ± 62.6	36.2 ± 31.1	0.31	
Δ SVA, mm	7.8 ± 31.6			2 ± 56.4			0.94
Odontoid–hip axis (OD-HA), °	1.2 ± 3.6	1.5 ± 2.4	0.67	1.8 ± 5.1	1.3 ± 4.1	0.92	
Δ OD-HA, °	0.3 ± 3.6			−0.5 ± 5.9			1.0

**Table 4:** Multinomial logistic regression for analysis of segmental lordosis dependence on different independent anatomical and surgical factors. Loss of lordosis (<0°) after surgery was set as the reference. Heteroskedastic variance structure was assumed by White’s test (*p* < 0.05).

	Factors	Regression Coefficient	*p*	Odds Ratio	95% Confidence Interval for OR	
					Lower Bound	Upper Bound
Lordosis gain 0–2°	Baastrup phenomenon	−5.247	0.179	0.005	0.000	11.033
	Grade of facet joint degeneration [17,18]	2.071	0.337	7.935	0.116	544.292
	BF vs. UF	−1.417	0.474	0.242	0.005	11.768
	Cage position (ventral vs. middle)	−0.084	0.961	0.919	0.030	27.756
	Cage height (all 8° oblique)	2.236	0.087	9.360	0.726	120.744
Lordosis gain >2°	Baastrup phenomenon	−0.169	0.850	0.845	0.147	4.855
	Grade of facet joint degeneration [17,18]	−0.874	0.304	0.417	0.079	2.208
	BF vs. UF	0.610	0.477	1.841	0.342	9.902
	Cage position (ventral vs. middle)	0.422	0.621	1.525	0.286	8.139
	Cage height (all lordotic 8°)	0.439	0.323	1.551	0.650	3.698

**Table 5:** Adverse events and postoperative complications. More than one event coincided in some cases.

Parameters	UF (n = 81)	BF (n = 23)	*p*
All cases in total, % (n)	25.9 (21)	34.8 (8)	0.28
Cases with surgical intraoperative adverse events, % (n)	21 (17)	21.7 (5)	0.57
Events of dural lesion on the cage insertion side (ipsilateral)	21 (17)	8.7 (2)	
Events of screw misplacement	3.6 (3)	4.3 (1)	
Events of screw dislocation/pullout by reposition/compression	2.4 (2)	8.7 (2)	
Events of intraoperative cage subsidence	1.2 (1)		
Cases with early surgical postoperative complications (up to 6 weeks postoperatively), % (n)	6.2 (5)	13.04 (3)	0.25
Events of epidural hematoma/surgical evacuation	1.2 (1)	4.3 (1)	
Events of lesion of the ipsilateral exiting root	3.6 (3)		
Events of aseptic superficial wound healing disorder (contralateral)		8.7 (2)	
Events of superficial wound infection (ipsilateral)	1.2 (1)		
Events of symptomatic contralateral sequester dislocation		4.3 (1)	
General intraoperative adverse events and early postoperative complications, % (n)	0	0	

## Data Availability

The datasets generated and/or analyzed in this study are not openly available due to reasons of sensitivity and are available from the corresponding author upon reasonable request. Data are located in controlled access data storage at the University Hospital of Cologne.

## Abbreviations

The following abbreviations are used in this manuscript:

ADH	Anterior disc height
ASA	General health status according to ASA grade
BF	Bilateral facetectomy
BMI	Body mass index
LLA	Lumbar lordosis angle
MI-TLIF	Minimally invasive transforaminal lumbar interbody fusion
NRS	Numeric rating scale
OD-HA	Odontoid–hip axis
PDH	Posterior disc height
PI	Pelvic incidence
PT	Pelvic tilt
SL	Spondylolisthesis
SLA	Segmental lordosis angle
SS	Sacral slope
SVA	Sagittal vertical axis
UF	Unilateral facetectomy
WHO	World Health Organization
